# A holistic view on disease‐modifying aspects, comorbidities, and contemporary neuroprotective approaches

**DOI:** 10.1002/nep3.70023

**Published:** 2025-12-31

**Authors:** Piotr Walczak, Shen Li, Xunming Ji, Johannes Boltze

**Affiliations:** ^1^ Center for Advanced Imaging Research, Department of Diagnostic Radiology and Nuclear Medicine University of Maryland Baltimore Maryland USA; ^2^ Department of Neurology and Psychiatry, Beijing Shijitan Hospital Capital Medical University Beijing China; ^3^ Beijing Institute of Brain Disorders Capital Medical University Beijing China; ^4^ Chinese Academy of Medical Sciences and Peking Union Medical College China; ^5^ University of Warwick School of Life Sciences Coventry UK

**Keywords:** Biomaterial, glial cells, ischemic stroke, neurodegeneration, neuroinflammation, neuronal differentiation, Parkinson's disease, postoperative delirium, sleep deprivation, spinal cord injury, stem cell therapy

Most conditions of the central nervous system (CNS) and the peripheral nervous system are not stand‐alone disorders but are modulated and influenced by other pathobiological processes. For instance, the impact of frequent comorbidities such as hypertension^1^ or dyslipidemia^2^ as factors contributing to and aggravating CNS diseases has been much better understood in recent years. Another prominent disease‐modifying factor is the gut microbiome which can exert both protective and detrimental effects on the nervous system in health and disease.^3^ An even better understanding of these factors will contribute to the development of individualized treatment approaches, a major research objective in the era of precision medicine.^4^ The current issue of *Neuroprotection* presents articles focusing on disease‐modifying factors or novel treatment approaches for a broad spectrum of conditions. This provides a wide perspective but also novel insights into disease mechanisms and targeted treatment approaches. Priority has been given to articles reporting, reviewing or meta‐analyzing clinical data as well as articles providing results of translationally relevant preclinical work.

Stem cell‐based therapies have been considered as a promising complementary therapeutic option to established treatment approaches for multiple neurological conditions—especially those for which only limited causal treatment options are available.^5^ Bou Najm and Alame review the therapeutic potential of stem cell‐based therapies on a wide spectrum of neurological disorders with a major yet not exclusive focus on adult stem cells and stem cell‐containing populations. While this is not a new topic in general, the authors specifically focus on clinical data obtained in pediatric patients. There is indeed a paucity of reviews providing such analyses, so the work by Najm and Alam provides a useful and new perspective. Although the pediatric studies reviewed are often early‐stage investigations and provide only preliminary findings or sometimes lack a randomized and controlled design, the observations made seem to confirm the overall good safety profile of stem cell‐based treatment that has been reported in previous studies enrolling adults. On the other hand, translational barriers that have been identified for stem cell‐based therapies in adult neurological patients also seem to be present in pediatric patient populations. A particularly interesting feature of the review is the inclusion of conditions which are rarely approached with stem cell‐based therapies such as epilepsy and autistic spectrum disorders.

Jiang and coworkers provide a review paper describing the relationship between Parkinson's disease (PD) and gallstones. This combination seems to be unusual at first glance but there is indeed a remarkably high incidence of gastrointestinal symptoms in early stages of PD. Microbial dysbiosis could contribute to the development of gallstone disease which in turn may have a detrimental impact on the intestinal microbiome, creating a vicious cycle. Moreover, there are interesting similarities between microbial dysbiosis in PD and gallstone disease. The review provides a detailed analysis of potential pathobiological relationships and disease mechanisms. The authors also provide an overview of the effects of probiotic interventions including gut microbiota transplantations in PD patients. A causal connection between PD and gallstone disease still needs to be established but the review presents several interesting working hypotheses that could be addressed in downstream research, especially by prospective cohort studies. The review finally describes how a deeper investigation of the relationship between gallstone disease and PD may potentially lead to the exploration of novel biomarkers that may aid early detection or even prevention of PD.

Neuroinflammation is a key feature and important therapeutic target in many CNS disorders.^6^, ^7^ Neuroinflammation is a complex phenomenon that requires well‐balanced multi‐target interventions. Network pharmacology is believed to be a promising tool to help unravel mechanistic interactions and to suggest meaningful therapeutic approaches.^8^ Zhang and co‐workers provide a bibliographic analysis of contemporary research on network pharmacology approaches in neuroinflammation research highlighting key contributing factors and most relevant therapeutic targets. There has been a steeply increasing number of publications reporting such approaches since 2020. Primary therapeutic targets are the NLR family pyrin domain‐containing 3 (NLRP3) inflammasome, apoptosis caused by neuroinflammation, and microglial cells as important neuroinflammatory mediators. Moreover, network pharmacology approaches have paved the way for multi‐target therapeutic strategies. The work by Zhang and colleagues provides a comprehensive description of a rapidly evolving field.

Macro‐ and microglial cells play important roles in the CNS steady state as well as under disease conditions, exerting both beneficial and detrimental effects. Thus, glial cells represent a promising therapeutic target since they are considered as both disease‐modifying elements and key effectors that potentially support interventional strategies.^9^ Olajide provides a concise yet comprehensive research highlight pointing out recent findings in the field which are presented in a review‐like style. A specific focus is set on factors and processes mediating the interaction between glial cells and neurons in neurodegenerative conditions, including mitochondrial transfer, exosomes, and glutamate level regulation. Glial cells also provide considerable metabolic support to neurons which increases resilience under pathological conditions. Although these beneficial glial cell actions are not sufficient to ultimately stop neurodegeneration in most conditions, augmenting their impact by targeted intervention may be a strong rationale for future neuroprotective approaches. These may also include the rebalancing of beneficial versus detrimental effects of glial cells under pathological conditions.

Traumatic spinal cord injury (SCI) is a severe condition very often leading to functional impairment and a significant loss of independence as well as quality of life in affected patients. Axons can regrow in principle and stem cells are present in small numbers in the spinal cord. However, attempts to use this endogenous regenerative potential have repeatedly failed since numerous pathological mechanisms such as the development of an impenetrable glial scar after SCI prevent a spontaneous or stem cell‐assisted regeneration of severed axon connections in the spinal cord.^10^ The use of biomaterials may provide spatial cues that have the potential to enhance the efficacy of endogenous, stem cell‐based regeneration in SCI. Fan and colleagues report results from a preclinical study investigating the impact of taxol‐containing collagen, a microtubule‐stabilizing agent that inhibits astrocytic differentiation but promotes neuronal differentiation of neural stem cells. Mice were treated with injections of collagen into the transection site. Collagen was either administered in its native form or contained taxol. Animals were followed up for 8 weeks. Mice treated with taxol‐loaded collagen showed increased differentiation of endogenous Nestin‐positive cells into neuronal phenotypes. A tissue bridge connecting both ends of the spinal cord transection was observed in mice treated with taxol‐loaded collagen and there was an improvement of electrophysiological parameters suggesting improved signal conduction across the spinal cord transection. The functionalized collagen also inhibited the formation of a glial scar at the site of injury. Although these results require confirmation in downstream research, as well as proof of true functional regeneration, this is a novel and promising approach that might eventually lead to better SCI treatment.

Poor sleep quality can contribute to degenerative CNS conditions and cognitive decline, but underlying mechanisms are still poorly understood. Mehta and colleagues conducted an explorative investigation to study transcriptional changes and differences in biomolecule expression in the locus coeruleus (LC) under rapid eye movement sleep deprivation. Transcriptional changes and resulting differences in protein concentrations were seen for dopamine β‐hydroxylase, tyrosine hydroxylase, and monoamine oxidase‐A. These enzymes are involved in noradrenaline metabolism. Specifically, dopamine β‐hydroxylase and tyrosine hydroxylase levels were increased whereas monoamine oxidase‐A levels decreased. These changes caused an overall increase in noradrenaline levels which is known to be associated with CNS conditions. Recovery from rapid eye movement sleep deprivation led to a normalization of gene and protein expression as well as noradrenaline levels. This research may lead to a better understanding of the consequences of poor sleep quality and related endogenous stress signaling that may be related to CNS disorders.

Surgical interventions can lead to relevant neurological complications. Some of these complications such as perioperative strokes or postoperative delirium (POD) are seen especially in older patients.^11^, ^12^ POD is a relevant morbidity factor as it impairs post‐surgical recovery and can have permanent consequences. It is known that certain anesthetics may increase POD risk. However, a reliable prediction of such events is not yet possible and thus a subject of ongoing research. Moreover, the mechanisms leading to POD are not yet fully understood. Dai and co‐workers conducted an in silico study using network toxicology and molecular docking analysis to investigate toxicity and mechanisms of isoflurane on POD. They identified eight potential genes that link isoflurane application to POD. This is meaningful because genetic factors may help to predict complex conditions that are based on multiple factors including genetic ones.^13^ The exact causal relationship between isoflurane use and POD may be investigated in future studies. To aid this, the study by Dai et al. also provides preliminary mechanistic insights. For instance, molecular docking analyses revealed binding affinity between isoflurane and several targets associated with dopaminergic signaling disruption and apoptosis. Thus, the work by Dai et al. provides valuable insight into isoflurane effects and its role in POD. It would now be interesting to also investigate the potential influence of more modern volatile anesthetics on POD incidence and severity. This may lead to improved anesthetic management of surgical patients to prevent perioperative complications.

The last article in the current issue of *Neuroprotection* is an interesting case report by Xiong and colleagues. They describe a patient presenting with acute hemichorea‐hemiballism and severe dysarthria. Diffusion‐weighted magnetic resonance imaging revealed multiple small infarcts in the right insula‐paraventricular and parieto‐occipital junction areas, as well as an occlusion of C1 segment of the right carotid artery. This is indeed a very rare constellation because hemichorea‐hemiballism is hardly seen as the initial manifestation of stroke and is usually caused by lesions situated in the contralateral basal ganglia and subthalamic nucleus. The authors describe a detailed diagnostic workup, applied treatments as well as the functional outcome which was favorable in this case. The authors further provide a detailed literature review and neuroanatomical analysis to present a potential explanation for this uncommon constellation. Thus, the case report is of good clinical interest.

The current issue of *Neuroprotection* presents a balanced mixture of reviews and review‐like articles as well as original contributions. They provide a good overview of aspects modifying the course of neurological diseases. As with the previous issue, the spectrum of topics is very wide, once again underlining the broad scope of *Neuroprotection*. Presented work provides some interesting mechanistic insight but also novel ideas and hypotheses to be confirmed or challenged by future research.

This is the last issue of *Neuroprotection* in 2025, and therefore a good opportunity to review the journal's development. We started the *Neuroprotection* with great hope and ambitions almost exactly 2.5 years ago, and the first issue was published in September 2023.^14^ Since then, we have received many manuscripts from the global neuroscience community. The number of submissions has steeply increased from year to year, and we were overwhelmed by the positive reception of *Neuroprotection*. The next year will bring even more advances as journal indexing in all major databases has been completed (e.g., Scopus) or will be completed very soon (e.g., Pubmed Central). Moreover, *Neuroprotection* will receive its debut journal impact factor in June 2026. These major milestones have been reached very quickly, taking almost the minimum time required. We wish to express our gratitude to all authors, reviewers, editorial board members and journal operating staff for their tremendous and continuous support that has made all this possible. We do look forward to embarking on an exciting new year, that will hopefully see even more outstanding articles being published in *Neuroprotection*. As a token of our gratitude, we will waive article processing fees in 2026 as well. We think that this is an inclusive way of serving and supporting the global neuroscience community and do look forward to submissions received in 2026.

## CONFLICT OF INTEREST STATEMENT

Xunming Ji, Piotr Walczak, and Johannes Boltze are Co‐Editors in Chief of *Neuroprotection*. Shen Li serves as an executive editor for the journal. All authors were blinded from reviewing or making decisions on the manuscript. The article was subject to the journal's standard procedures, with peer review handled independently of the Co‐Editors in Chief and their research groups. Piotr Walczak is a founder and holds equity in IntraArt, LLC and Ti‐com, LLC.

## ETHICS STATEMENT

Not applicable.

## Data Availability

Data sharing is not applicable to this article as no new data were created or analyzed in this study.
